# Second Malignancy Masquerading as Recurrence of Neuroendocrine Tumor

**DOI:** 10.1210/jcemcr/luad127

**Published:** 2024-01-27

**Authors:** Ananda Mohan Chakraborty, Neha Bhardwaj, Ashu Rastogi, Sanjay Kumar Bhadada

**Affiliations:** Department of Endocrinology, PGIMER, Chandigarh 160012, India; Department of Pathology, PGIMER, Chandigarh 160012, India; Department of Endocrinology, PGIMER, Chandigarh 160012, India; Department of Endocrinology, PGIMER, Chandigarh 160012, India

**Keywords:** mucoepidermoid carcinoma, second malignancy, post radiotherapy malignancy, ectopic Cushing syndrome

## Abstract

Neuroendocrine tumors (NETs) may mimic many endocrine syndromes, including Cushing syndrome (CS) secondary to ectopic ACTH secretion. Radiotherapy (RT) is often used as adjuvant therapy for such persistent or recurrent NETs. However, RT may predispose a susceptible person to a second malignancy. Here, we reported the story of a 37-year-old male, who presented with progressive weight loss, bone pain, and shortness of breath in the emergency department. He was diagnosed with CS secondary to a carcinoid tumor in the bronchopulmonary tree a decade previous and underwent total bilateral adrenalectomy. He also underwent lobectomy, and subsequent RT for a primary NET and was in clinical remission. His presenting symptoms were considered a recurrence of pulmonary NETs. However, the biopsy suggested high-grade mucoepidermoid carcinoma (MEC). MEC of the lung is a rare tumor with a prevalence of <1% of all lung malignancies. MEC of the lung after RT for bronchial NET-causing ectopic CS has not yet been reported in the literature. The present patient did not survive despite achieving remission of CS and primary tumor because of the aggressive second malignancy attributed to RT, which was given for the primary tumor.

## Introduction

Neuroendocrine tumors (NETs) may mimic many endocrine syndromes including Cushing syndrome (CS) secondary to ectopic ACTH secretion commonly known as ectopic Cushing syndrome (ECS). ECS often becomes challenging to manage as primary tumor-causing ECS may remain elusive. Towering, high ACTH and cortisol levels in those patients with ECS cause derangement in metabolic and cardiovascular functions, psychiatric problems, and overwhelming infections and often requires total bilateral adrenalectomy (TBA) for disease remission [1]. Patients with TBA often pose a great challenge because iatrogenic hypocortisolemia may mask the progression and recurrence of a primary NET. Patients will remain mostly asymptomatic until the local disease produces significant local symptoms. These elusive NETs often require surgery and/or radiotherapy (RT) for control of primary pathology even after TBA. RT is considered the most plausible option for surgically nonamenable tumors [2].

RT, however, is considered a double-aged sword because in some instances it is considered as the only viable option, especially in certain cryptogenic and recurrent NETs; on the other hand, there is a potential risk of RT-induced malignancies (RIM). The reported second malignancies after RT include sarcomas, carcinomas, leukemias, and mesotheliomas [3].

Mucoepidermoid carcinoma (MEC) primarily affects the salivary gland located in the head and neck region. Mucous and serous glands in the respiratory tract rarely produce neoplasms that are histologically identical to neoplasms derived from the salivary glands [4]. MEC accounts for approximately 0.1 to 1.0% of all lung cancers. Pulmonary MEC is a rare malignant tumor first described by Smetana in 1952 [5].

Pulmonary MECs usually arise in the submucosa of the large bronchi and mainly consist of the mucous-secreting cell, squamous cell, and intermediate cell types [5]. They are classified histologically as low-grade and high-grade tumors to predict optimum outcomes. A long asymptomatic lag time before diagnosis always poses a challenge in the management of such RIM, especially if they arise at the same location as the primary tumor.

## Case Presentation

A 37-year-old male presented at the emergency department with progressive weight loss, bone pain, shortness of breath, and nonproductive cough. He had generalized plethora, weight gain, broad violaceous striae over the abdomen and limbs, nail and skin pigmentations, proximal muscle weakness, hypertension, and bilateral avascular necrosis of the femur at the time of the preceding presentation a decade ago. His cortisol dynamics (high-dose dexamethasone suppression test was not suppressible and nonstimulable ACTH and cortisol on desmopressin stimulation test) and negative contrast-enhanced magnetic resonance imaging of sella was favoring ECS. Subsequently, contrast-enhanced computed tomography of the thorax revealed a left lingular lobe space-occupying lesion, and fine-needle aspiration cytology confirmed it as a NET of the bronchopulmonary tree. A diagnosis of CS secondary to an ectopic ACTH-producing carcinoid tumor of the bronchopulmonary tree was retained at that time. Because of the high cortisol burden (8 Am cortisol 1263 nmol/L [45.78 µg/dL], 11 Pm cortisol 1365 nmol/L [49.48 µg/dL], ACTH 1263 pg/mL [normal range, 7.5-63 pg/mL]; the detection limit was 1.5-2000 pg/mL), and acuteness of presentation, he underwent TBA. After TBA, he was put on a replacement dose of glucocorticoid and mineralocorticoid. With the remission of CS and achievement of hypercortisolism, the bronchopulmonary tree carcinoid was troubling the patient because of progressive local symptoms and pigmentation. He underwent resection of the tumor, which was suggestive of an atypical carcinoid with necrosis and high mitotic index. He underwent local RT because the resection margin was positive for the tumor (fractionated external beam radiotherapy, 40 Gray in 20 fractions).

**Figure 1.**
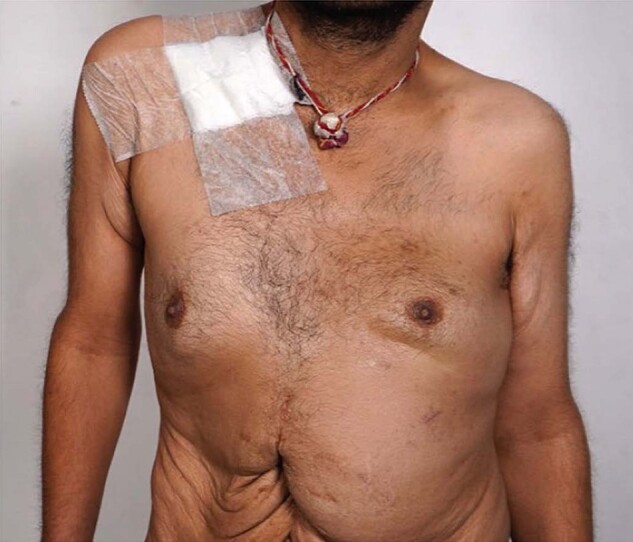
The clinical image of the index patient shows shoulder drooping rib crowding, scattered pigmentation, and abdominal scar.

He had clinical and radiological remission subsequently for at least 5 years. He was lost to follow-up for 4 years. After almost a decade after the initial presentation, he presented with acute decompensation at the emergency department during his recent visit. Clinical examination revealed multiple lymph nodes at the anterior aspects of the neck and supraclavicular locations on both sides. Respiratory system examination revealed left-sided fibrosis of the lung with crowded ribs, shoulder drooping, restricted movement with a dull note, and reduced breath sounds. There was significant bony tenderness over the ribs, sternum, and thoracic vertebrae. Other systems examinations were not contributory (Fig. 1).

## Diagnostic Assessment

**Figure 2.**
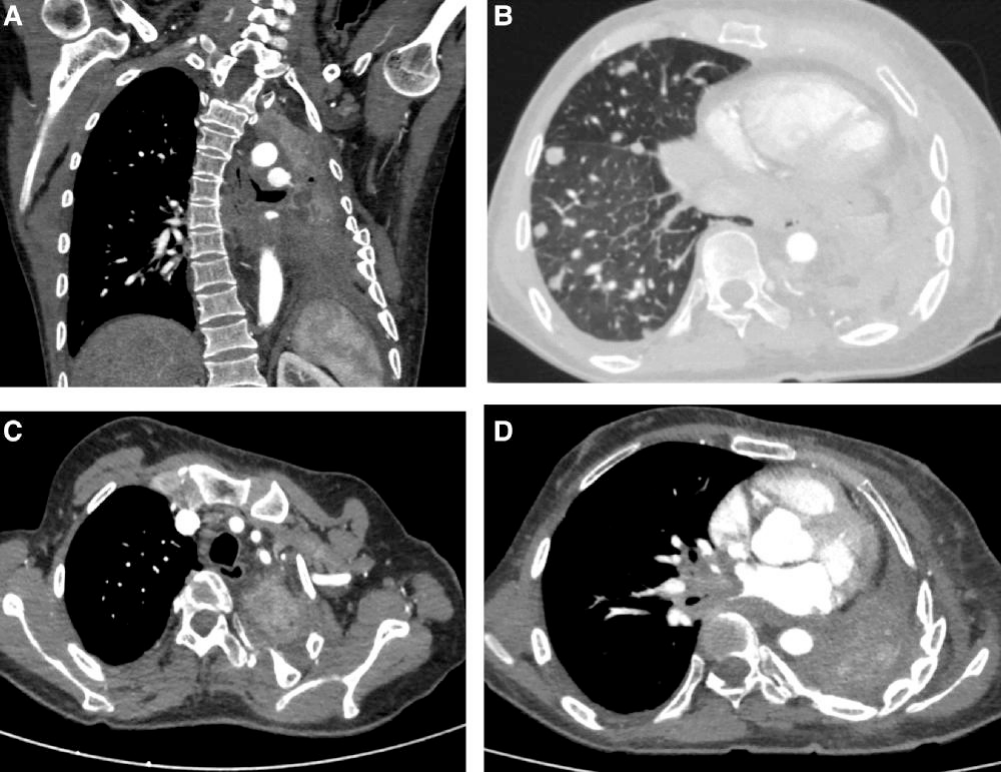
Contrast-enhanced computed tomography of the thorax revealed the collapse of the left lung with fibrotic changes (A), multiple nodules in the right side of the lung with mediastinal lymphadenopathy (precarinal, right upper, and lower paratracheal, with right hilar being largest at1.5 × 1.6 cm) (B), heterogeneously enhancing nodular lesions in the extrapleural space on the left side extending along the intercostal nerves in the ninth, 10th, 11th, and intercostal space on the left side, the largest measuring 9.6 × 9.5 mm (C and D).

His blood biochemistry and hormone profile were suggestive of anemia, elevated bone turnover marker, and high-normal ACTH (Table 1). Contrast-enhanced computed tomography of the thorax was suggestive of left lobectomy status, complete collapse along with multiple nodules on the right side, and multiple locoregional lymph nodes suggestive of metastatic disease (Fig. 2A-D).

**Table 1.** Biochemical and hormonal profile of index patent

Laboratory tests	Observed value	Reference range
Complete hemogram	Hb 7.8 g/dL, TLC: 9.2 × 10^9^/L, Neutrophils 65%, Lymphocytes 23%, Platelet: 95 × 10^9^/L	12-16 g/dL 4-11 × 10^9^/L 150-450 × 10^9^/L
Renal function test	Urea: 46 mg/dL (16.42 mmol/L) Creatinine: 1.34 mg/dL (118.4 µmol/L) Na: 136 mmol/L K: 4.2 mmol/L	10-50 mg/dL (3.57-17.85 mmol/L) 0.5-1.2 mg/dL (44.2-106 µmol/L) (135-145 mmol/L) (3.5-5 mmol/L)
Liver function test	Bilirubin: 1.01 mg/dL (17.27 µmol/L) ALT: 67 U/L AST: 74 U/L ALP: 176 U/L	0.2-1.2 mg/dL (3.42-20.52 µmol/L) (2-41 U/L) (2-40 U/L) (42-128 U/L)
8 Am Cortisol ACTH T4 T3 TSH	20.55 µg/dL (567 nmol/L) (On hydrocortisone supplement) 58 pg/mL (12.76 pmol/L) 7.8 µg/dL (100.3 nmol/L) 1.34 ng/mL (0.020 nmol/L) 4.5 µIU/mL	6.19-19.42 µg/dL (171-536 nmol/L) 7.5-63 pg/mL (1.65-13.86 pmol/L) 4.8-12.7 µg/dL (61.7-163.4 nmol/L) 0.8-2 ng/mL (0.01-0.03 nmol/L) 0.27-4.2 µIU/mL
P1NP CTX Chromogranin A	56 ng/mL 1356 pg/mL 24 ng/mL	12.46-83.47 ng/mL 225-936 pg/mL < 39 ng/mL
Calcium Phosphate Magnesium Vitamin D	9.8 mg/dL (2.45 mmol/L) 3.2 mg/dL (1.03 mmol/L) 1.2 mg/dL (0.49 mmol/L) 14 ng/mL (34.9 nmol/L)	8-10.2 mg/dL (2-2.55 mmol/L) 3.5-5.5 mg/dL (1.13-1.77 mmol/L) 1.8-2.4 mg/dL (0.7-0.9 mmol/L) 20-40 ng/mL (49.9-99.8 nmol/L)
HbA1c FPG PPPG	5.4% 94 mg/dL (5.217 mmol/L) 126 mg/dL (6.993 mmol/L)	(<6.5%) < 126 mg/dL (6.993 mmol/L) < 200 mg/dL (11.1 mmol/L)
Blood culture Urine culture	Negative for any growth Negative for any growth	

Abbreviations: ALP, alkaline phosphatase; ALT, alanine aminotransferase; AST, aspartate aminotransferase; CTX, C-terminal cross-linked telopeptide of type I collagen; FPG, fasting plasma glucose; Hb, hemoglobin; P1NP, procollagen type I N-terminal pro-peptide; PLT, platelets; PPPG, postprandial plasma glucose; TLC, total leukocyte count.

**Figure 3.**
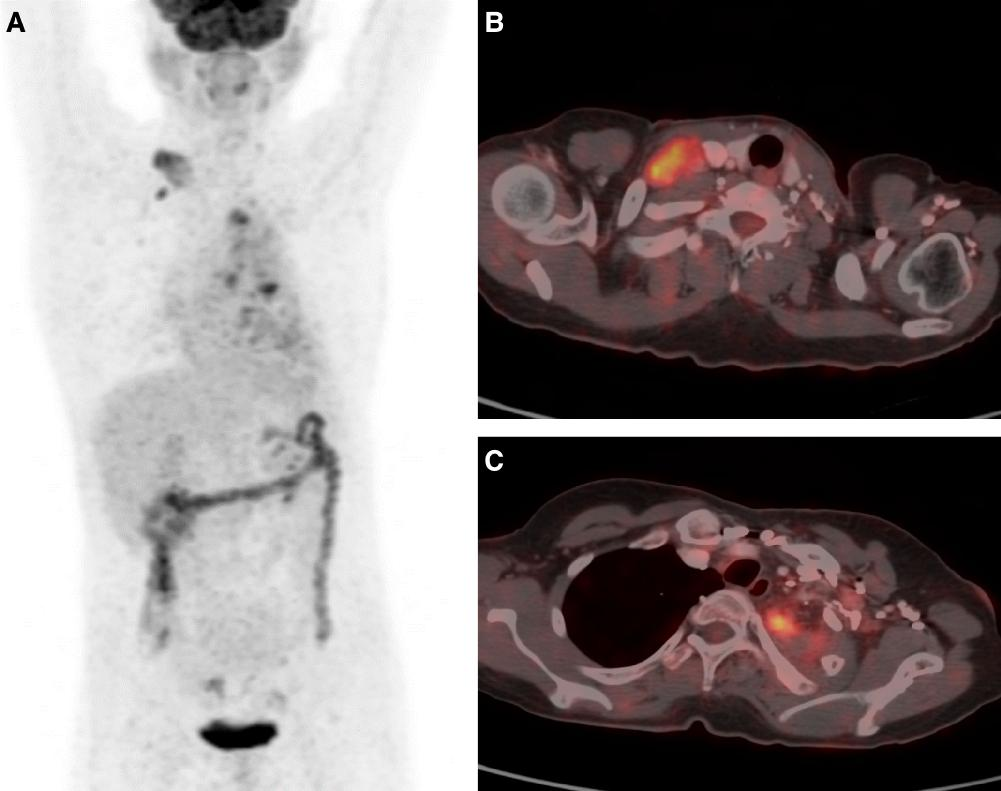
18 F Fluro deoxy glucose positron emission tomography computed tomography (18F FDG PET-CT) showing a whole-body planer image with normal tracer uptake in the brain and bladder, with some uptake in the cervical and thoracic regions (A). A few pleural-based, ill-defined lesions with a maximum standard uptake value (SUVmax) of 7.7 in the apex of the left lung and multiple nodular soft-tissue densities of varying sizes on the left lung (B). Tracer-avid enlarged right supraclavicular lymph nodes, the largest being 2.0 × 3.4 cm with an SUVmax of 8.2. Mildly tracer-avid left supra- and infraclavicular lymph nodes. Tracer-avid subcentimetric to centimetric lymph nodes at paratracheal left posterior intercostal locations (C).

**Figure 4.**
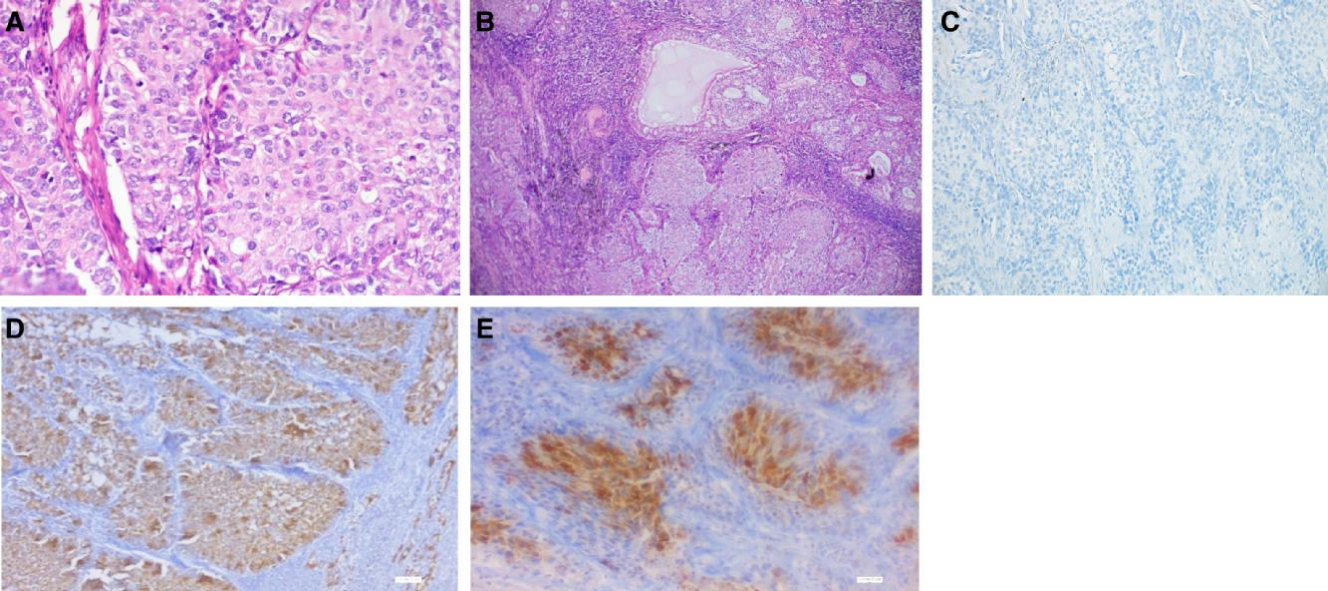
Histopathology and immunohistochemistry of lymph node biopsy specimen (high-power field and low-power field) showed solid nests and lobules of tumor cells infiltrating the lymph node (A). The eosinophilic intermediate cells are mixed with mucous cells floating in extracellular mucin (B). The tumor cells are negative for ACTH immunostaining (C). The tumor cells are positive for MUC5AC and p40 (D and E).

18F Fluro deoxy glucose positron emission tomography computed tomography was suggestive of metastatic disease (Fig. 3A-C). Upon suspicion of recurrence of NET, he underwent a lymph node biopsy from the right supraclavicular lymph node. However, the biopsy was suggestive of high-grade MEC (Fig. 4A-E). Finally, a diagnosis of metastatic high-grade MEC of the lung was made.

## Treatment

He was offered palliative radiotherapy, first at the lung and mediastinum and then to the right supraclavicular lymph node.

## Outcome and Follow-up

He succumbed to his illness 2 months following diagnosis.

## Discussion

Mucoepidermoid carcinoma of the lung is a rare tumor with a prevalence of <1% of lung malignancies, with no definitive etiology [6]. A tumor is considered as RIM if it fulfills modified Cahan's criteria [7].

Carcinoma and leukemia are commonly seen in low-dose radiation recipients and sarcoma is more common in high-dose radiation recipients [3, 8]. Other nonmalignant conditions documented post-RT are pulmonary and cardiac fibrosis. Sometimes they produce overlapping symptoms. MEC of the lung secondary to RT has been reported previously in the literature in the background of Wilms' tumor [9] but has never been reported in a patient with bronchial NET causing ectopic CS. Primary MEC has a varied age of presentation ranging from the third to the seventh decade with clustering around fifth decade in the literature. The age of presentation of secondary MEC might be determined by the age of the radiation insult. Surgery is considered as the primary therapy for MEC followed by RT. The outcome of such therapy is determined by tumor stage and pathological grade (Table 2) [10]. Long-term follow-up of patients with NETs is difficult because the lack of a sensitive biochemical surrogate of tumor progression and frequent radiological follow-up might be cumbersome from the cost of investigation and patient compliance. In our patient, disease presentation after TBA was late and almost was not found because symptoms related to locoregional recurrence were the only discernible clinical manifestation, which was partly masked by our patient’s radiation-induced pulmonary fibrosis. Radiology was not proficient enough to detect the disease early before an 18F fluoro deoxy glucose positron emission tomography computed tomography scan was done. Even after the reconciliation of symptoms and signs, our patient was thought to have NET recurrence only until his biopsy showed that he had MEC, which qualifies as RIM. Therapy for most of the cases of pulmonary MEC as per the literature was surgery. Considering our patient's advanced stage (stage IV), we offered him palliative RT at the lung bed and lymph nodes.

**Table 2.** Demography treatment modality and outcome of primary pulmonary MEC reported in the literature

Author	Year	Number of cases	Age	Gender	Treatment	Survival data
Jun-Jie-Xi (Xi et al, 2012)	2004-2011	21 Low grade: 17 High grade: 4	Mean Age 43.4	M 10 F11	Surgery In all 21 cases	The follow-up period for the 16 low-grade tumor cases ranged from 5 to 77 months (mean, 46.6 months). The average age of the patients was 38.8 years. Among these patients, 15 survived and 1 died. The follow-up period for the 4 high-grade tumor cases ranged from 23 to 65 months (mean, 41.5 months). The average age of the patients was 62.0 years. Among these patients, 3 died and 1 survived.
Zhu et al (Zhu et al, 2013)	2013 (2001-2013)	69 Low grade 45; intermediate grade 11; high grade 13	Median age 47.5 (7-73)	M 38 (55.1) F 31 (44.9)	Surgery 66 Chemotherapy 3 Radiotherapy 4	Stage I-IIA: 5 years’ survival: 90.8% IIB-IV: 5 years’ survival: 54% Grade Low to intermediate: 5 years’ survival: 95% High grade: 5 years’ survival: 41.7%
Komiya et al (Komiya et al, 2017)	2016 (1973-2012)	423 Low grade 226; high grade 73, unknown 124	≥39 years 130 cases (30.7%) 40-69 years 219 cases (51.8%) ≥70 years 74 cases (17.5%)	M 232 (54.8) F 191 (45.2)	Surgery alone 274 Surgery + radiation 30 Radiation alone 64 Neither 55	Stage Localized: 5 years’ survival: 97% Regional: 5 years’ survival: 56.9% Distant: 5 years’ survival: 8.2% Grade Low grade: 5 years’ survival: 90.6% High grade: 5 years’ survival: 28.6%
Salem et al (Salem et al, 2017)	2017	16 Low grade 14; high grade 2	Median age 40.4 (7.4-82.9)	M 7 (43.6) F 9 (56.3)	Surgery 14 Radiation 6 Chemotherapy 3	Median follow-up, months 40.7 (1.7-120.1) Died: 3 (18.8) Alive: 13 (81.2)

Abbreviations: F, female; M, male.

ECS has an overall poor prognosis either because of elusive primary or metastatic disease. Though our patient became asymptomatic after TBA for hypercortisolism and lobectomy followed by RT for bronchial carcinoid, he did not survive despite achieving remission of the primary tumor because of the aggressive RIM.

Though survival data for primary MEC are variable, overall survival depends on tumor grade and stage. High-grade tumors and advanced stage at presentation are associated with poor survival ranging from <1% to 50% in the literature. Because pulmonary MEC as RIM is extremely rare, survival data are not readily available.

We find this case interesting because pulmonary MEC as RIM in a background of NET presenting as ECS has not been reported in the literature previously, and RIM masqueraded as a recurrence of the primary tumor until histopathology and immune histochemistry reports were available.

This is the first case of pulmonary MEC following RT for bronchial carcinoid presenting 10 years after radiation exposure. Though recurrence of primary malignancy is always a possibility, RIM should also be considered as a possible differential in such a scenario.

## Learning Points

- Diagnosis and management of ectopic Cushing syndrome can be challenging and, on some occasions, require total bilateral adrenalectomy.
- Long-term follow-up of neuroendocrine tumors is difficult because of the lack of sensitive biochemical surrogates, the cost of investigation, and the lack of compliance.
- Radiotherapy-induced malignancies arising in the same field of previous disease are often confused with tumor recurrence and cause potential delays in diagnosis and therapy.

## Contributors

All authors made individual contributions to authorship. A.M.C. and A.R. were involved in patient care and clinical diagnosis. N.B. was involved in the histopathological diagnosis. S.B. was involved in scientific decision-making and content modification.

## Data Availability

Some or all datasets generated during and/or analyzed during the current study are not publicly available but are available from the corresponding author upon reasonable request.

## Abbreviations

CS: Cushing syndrome
ECS: ectopic Cushing syndrome
MEC: mucoepidermoid carcinoma
NET: neuroendocrine tumor
RT: radiotherapy
TBA: total bilateral adrenalectomy
